# GenoCore: A simple and fast algorithm for core subset selection from large genotype datasets

**DOI:** 10.1371/journal.pone.0181420

**Published:** 2017-07-20

**Authors:** Seongmun Jeong, Jae-Yoon Kim, Soon-Chun Jeong, Sung-Taeg Kang, Jung-Kyung Moon, Namshin Kim

**Affiliations:** 1 Personalized Genomic Medicine Research Center, Division of Strategic Research Groups, Korea Research Institute of Bioscience and Biotechnology, Daejeon, Korea; 2 Department of Biological Sciences, KRIBB School, Korea University of Science and Technology, Daejeon, Korea; 3 Bio-Evaluation Center, Korea Research Institute of Bioscience and Biotechnology, Cheongju, Chungbuk, Korea; 4 Department of Crop Science and Biotechnology, Dankook University, Cheonan, Chungnam, Korea; 5 National Institute of Crop Science, Rural Development Administration, Jeonju, Jeonbuk, Korea; UMR-S1134, INSERM, Université Paris Diderot, INTS, FRANCE

## Abstract

Selecting core subsets from plant genotype datasets is important for enhancing cost-effectiveness and to shorten the time required for analyses of genome-wide association studies (GWAS), and genomics-assisted breeding of crop species, etc. Recently, a large number of genetic markers (>100,000 single nucleotide polymorphisms) have been identified from high-density single nucleotide polymorphism (SNP) arrays and next-generation sequencing (NGS) data. However, there is no software available for picking out the efficient and consistent core subset from such a huge dataset. It is necessary to develop software that can extract genetically important samples in a population with coherence. We here present a new program, GenoCore, which can find quickly and efficiently the core subset representing the entire population. We introduce simple measures of coverage and diversity scores, which reflect genotype errors and genetic variations, and can help to select a sample rapidly and accurately for crop genotype dataset. Comparison of our method to other core collection software using example datasets are performed to validate the performance according to genetic distance, diversity, coverage, required system resources, and the number of selected samples. GenoCore selects the smallest, most consistent, and most representative core collection from all samples, using less memory with more efficient scores, and shows greater genetic coverage compared to the other software tested. GenoCore was written in R language, and can be accessed online with an example dataset and test results at https://github.com/lovemun/Genocore.

## Introduction

Selecting core subsets from large collections is an effective strategy to characterize and utilize genetic resources of crop plants without the requirement of sampling the entire population. This concept was first proposed by Frankel *et al*. to select a subset of the data that is representative of the whole resource, by removing redundant samples and maximizing genetic diversity [1]. Several methods have been developed and recently implemented for core sample selection, such as MSTRAT [2], PowerCore [3], Core Hunter [4], and Core Hunter II [5].

MSTRAT is a local search method that is based on a maximization strategy to maximize the richness of samples [2]. It randomly divides the entire sample into two groups, retains accessions to meet the greatest increase diversity criterion, and repeats the process until 30 iterations are completed or the richness no longer increases. Core Hunter I and II are based on an advanced stochastic local search algorithm implemented by Java [4–5]. Core Hunter II uses a mixed replica search method that is based on several search methods, including LR Semi Replica, Local Search Replica, Tabu Search Replica, and Simple Monte Carlo Replica. The first step in this process involves arbitrary selection of samples to shorten the execution time in common with MSTRAT. However, application of the default runtime option can vary greatly in each run in the case of a large dataset, which makes it difficult to decide on a key subset. Kim *et al*. developed PowerCore using a modified heuristic search algorithm, A* algorithm, based on graph theory, which extracts a subgraph while minimizing elements and paths [3]. This program gives similar results to those obtained with MSTRAT and Core Hunter, but PowerCore cannot load a large dataset.

In recent years, the numbers of samples and markers available for investigating genetic diversity has increased to thousands and millions, respectively, owing to the development of new high-throughout technologies such as 1,536 SNP chip of Illumina GoldenGate assay [6], Illumina MaizeSNP50 BeadChip [7], Axiom Soybean Genotyping Array [8], high-density rice array (HDRA, 700k SNPs) [9], Wheat 820k genotyping Array [10], and Axiom Wheat Breeder’s Genotyping Array [10]. In order to effectively analyze these datasets with many samples, a high-performance system and efficient algorithm are required. Some methods are more focused on analysis of rare alleles than on polymorphisms in order to compensate for the lack of markers, but rare variants are often removed by filtering according to the minor allele frequency, which is common in genome-wide association studies. We are more focused on common alleles than rare alleles, because most of features of interests are complex traits. We concentrated on common alleles to represent the entire sample at best, which helps to increase the genetic coverage.

In this paper, we present GenoCore, which is implemented in R (version 3.2.5), as a new method to select a core collection using modified statistical measures related to genetic allele coverage and diversity. By selecting samples with these measures, it is possible to quickly cover the entire samples, use less memory, and obtain a consistent final core subset. To compare GenoCore with other programs (MSTRAT, Core Hunter II, and PowerCore), we applied example datasets to each program.

## Results

### Dataset

For comparison, we applied GenoCore, MSTRAT, Core Hunter, and random sampling to wheat [10], 1.5k rice [6], and 700K rice [9] datasets, respectively. Table 1 provides the following key information of each dataset: single nucleotide polymorphism (SNP) chip name, marker, and the number of samples. Datasets of various sizes were tested to check the software for practicability, computation time, and accuracy, so that the optimal software can be chosen that offers the best solution for addressing the core collection problem.

**Table 1:** Datasets.

Dataset	SNP Chip	# of markers	# of samples
Rice 1.5K	Illumina GoldenGate Assay	1,536	395
Wheat	Affymetrix Axiom 35K SNP array	35,143	556
Rice 700K	High Density Rice Assay 700K SNPs	700,001	1,108

Since there is a dataset loading problem, GenoCore is the only software that can incorporate all datasets, and PowerCore can only read the 1.5k rice dataset; the other software programs cannot load a high-density SNP-chip such as the 700k rice dataset. We set the default options in GenoCore to a delta value of 0.01% and a coverage value of 99%. Since the first step of Core Hunter and MSTRAT is random sampling, no difference exists from randomly selected samples. Therefore, we increased the operation time using the runtime option for each method including random selection. The number of subsets from random sampling strategies is equal or similar to the number of subsets extracted from GenoCore. We analyzed the performance of the software using two computers, one running Windows 10 (Intel Core i5-3570K, 3.4 GHz CPU, 4 GB memory) for PowerCore, and the other running CentOS 6 (64 core AMD Opteron Processor 6380, 2.5 GHz, with 256 GB of main memory) for Core Hunter, MSTRAT, and GenoCore.

### Comparison

We compared the coverage (CV) [3], modified Rogers (MR) value [11], minimum MR value, Shannon diversity index (SH) [12], and required memory of GenoCore to those of MSTRAT, PowerCore, Core Hunter, and random sampling. Each of CV, MR and SH is defined by

$$CV=\frac{1}{m}\sum_{i=1}^{m}\frac{G_{C_{i}}}{G_{E_{i}}},\;SH=-\sum_{i=1}^{m}p_{i}\;\ln\;p_{i}$$

$${MR}_{xy}=\frac{1}{\sqrt{2m}}\sqrt{\sum_{i=1}^{m}\sum_{j=1}^{n_{i}}{\left(p_{xij}-p_{yij}\right)}^{2}}$$

Here, *m* is the number of markers, *p*_*xij*_ is the relative frequency of the *j*-th allele at the *i*-th locus for sample *x*, *p*_*i*_ is the relative reference allele frequency, and $G_{C_{i}}$ and $G_{E_{i}}$ are the number of genotype classes for core collection *C*_*i*_ and entire collection *E*_*i*_. Genotype class is the set of possible genotypes of a marker for all samples.

The software with superior core collection would show a higher genetic diversity (SH), coverage (CV), and genetic distance (MR), and use less hardware and execution time for improved efficiency.

**Fig 1:**
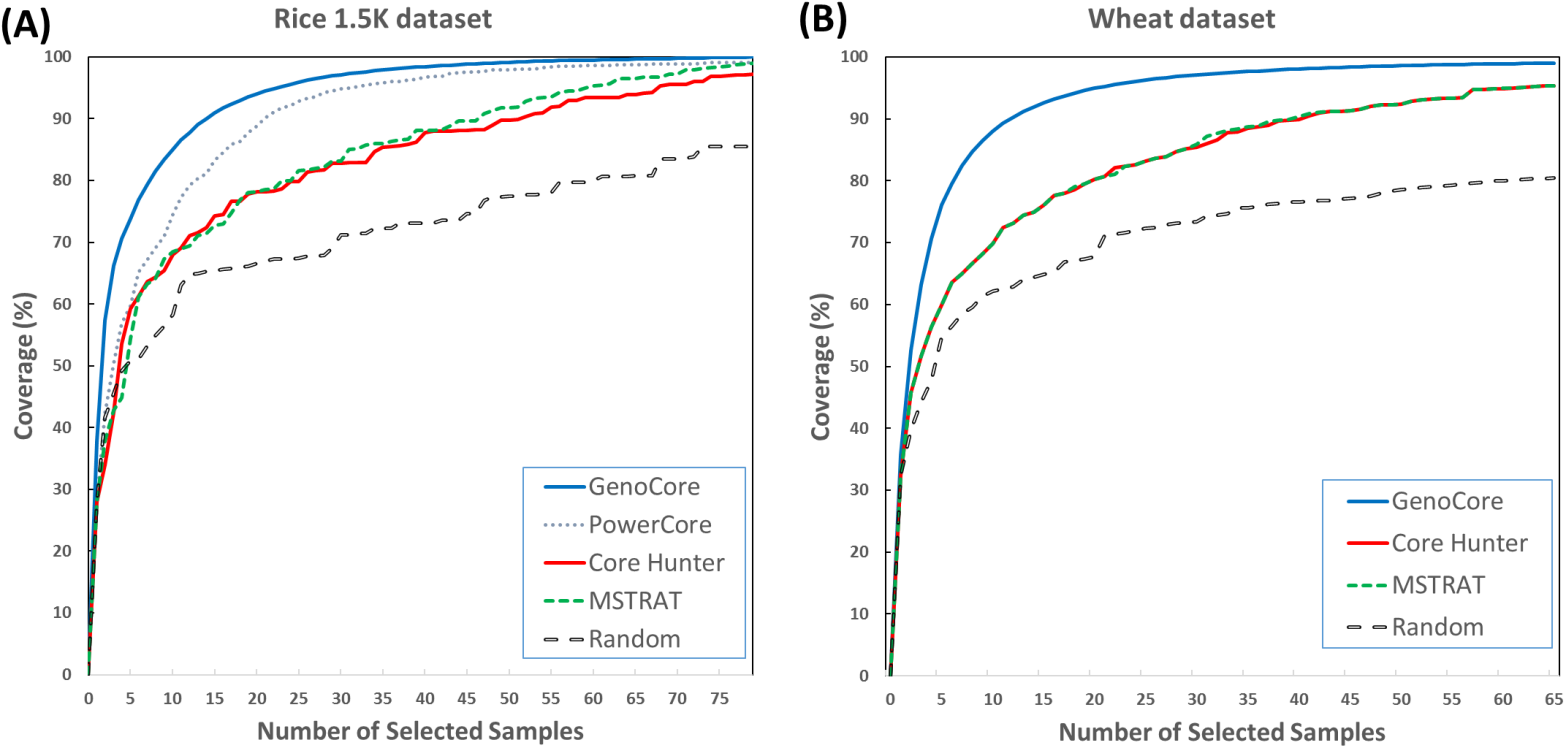
Increase in coverage values versus number of selected samples for each software. (A) Rice 1.5K dataset, (B) wheat dataset.

**Fig 2:**
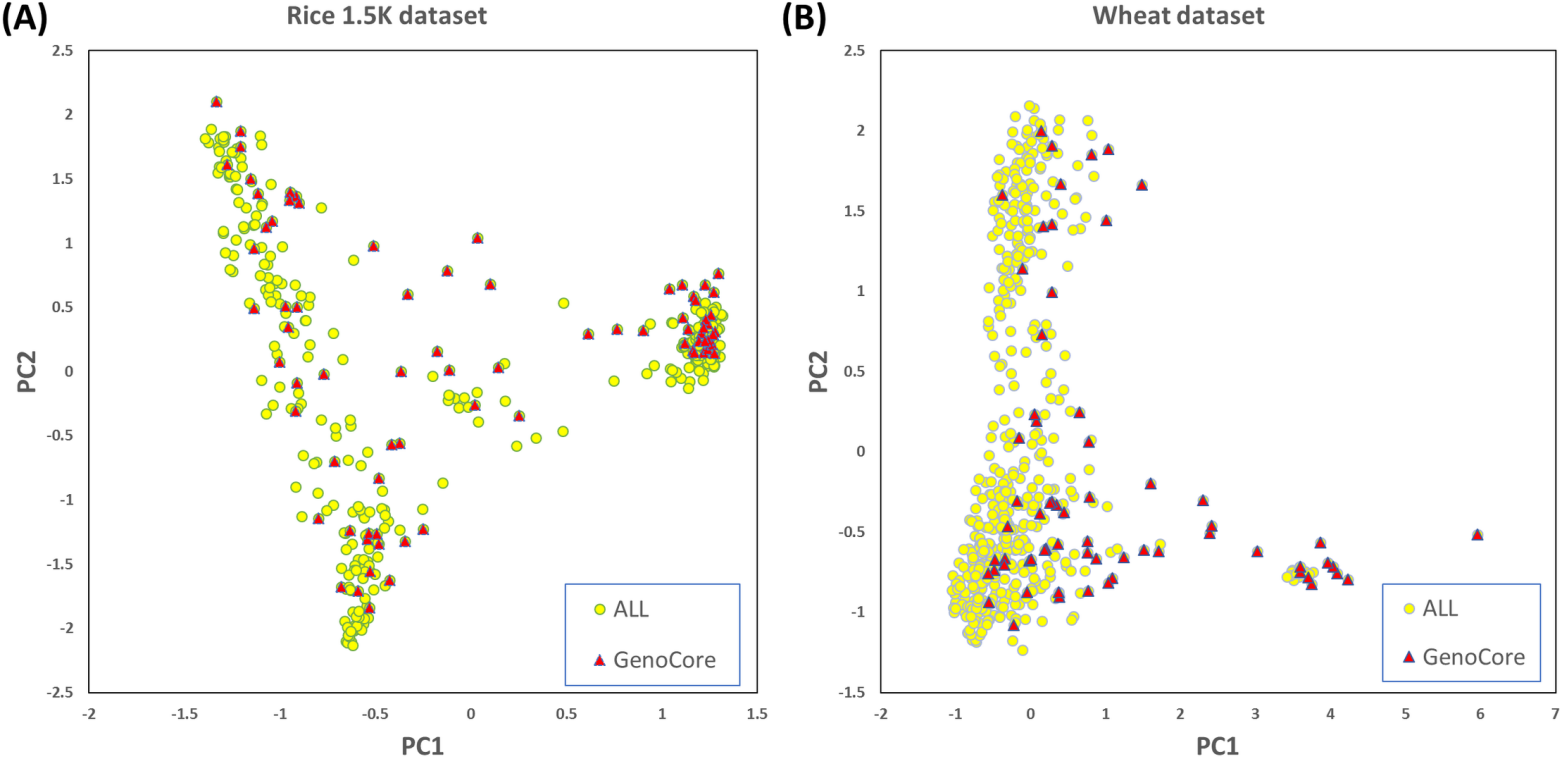
Principal component analysis. (A) Rice 1.5K dataset, (B) wheat dataset.

**Fig 3:**
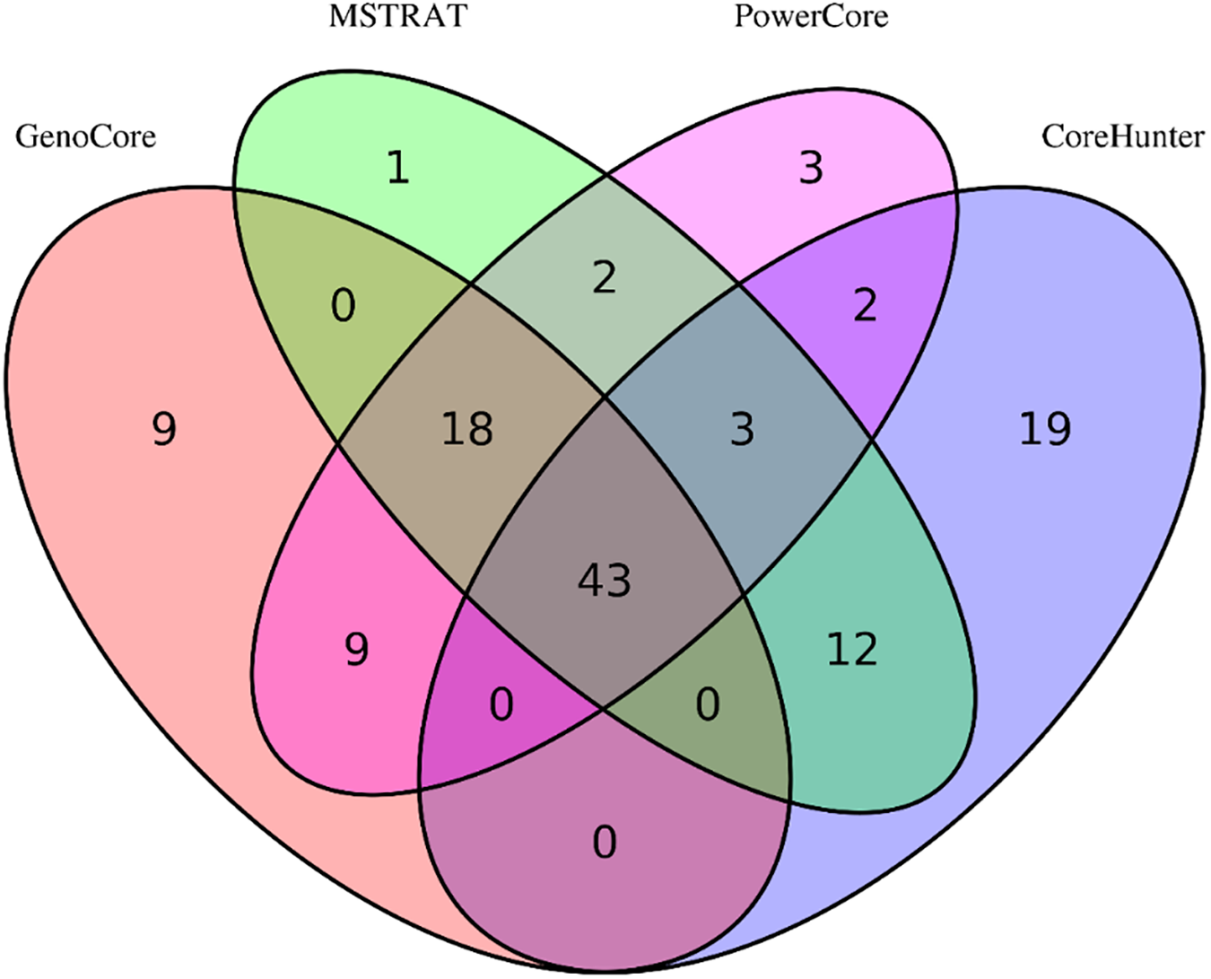
Venn diagram (rice 1.5K dataset).

**Fig 4:**
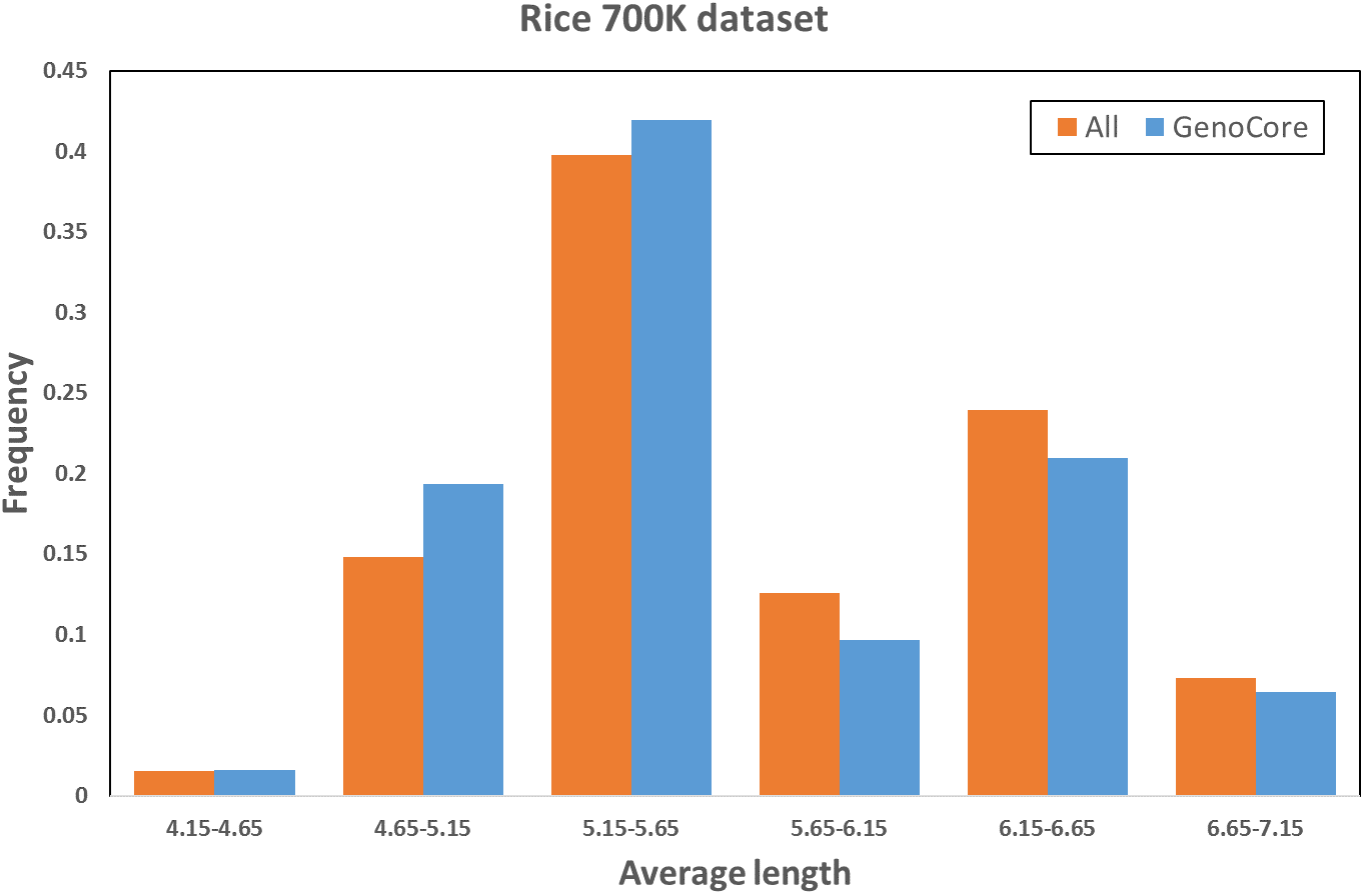
Phenotype density (rice 700K dataset).

Fig 1 denotes that the cumulative genetic coverages versus the number of selected samples in the 1.5k rice (Fig 1A) and wheat (Fig 1B) datasets. This means that core subset by GenoCore definitely covers the entire samples in fastest time compared with the other methods. GenoCore selected the core subset that achieved exactly or closest to 100% coverage, greater or similar values of MR and SH, and the minimum MR compared to the other methods, as shown in Tables 2 and 3. In particular, GenoCore showed a large difference in the minimum MR distance for all datasets, indicating that our method does not select similar genotype samples. Principal component analysis was conducted to show the distribution of the most informative variables for the population and location of core samples. Selected core samples by GenoCore were evenly spread across the population for principal components, PC1 and PC2, for the 1.5k rice (Fig 2A) and wheat (Fig 2B) datasets. We constructed a Venn diagram to show the common part of the core collection lists obtained from all software for the rice 1.5k dataset (Fig 3). Fourty-three samples were included for all methods, and 9 samples were uniquely selected in GenoCore. Fig 3 indicates that subsets from GenoCore, MSTRAT, and PowerCore share 61 samples (76%), but not with Core Hunter (54%). It is believed that diversities from genotypes will also represent diversities from phenotypes. Thus if we select the most diverse samples from population, distribution of phenotypes will be very similar to a population. Only our method could select the core collection for the 700k rice dataset (Table 4). Fig 4 shows that the entire and core subsets had a similar phenotypic distribution. Hence, GenoCore will represent subsets from reasonable genotypes and phenotypes diversities.

**Table 2:** Core collection results (rice 1.5K dataset).

Software	# of samples	MR	Min. MR	SH	CV
GenoCore	**79**	0.63968	**0.31785**	7.7815	**100**
Core Hunter	**79**	**0.64239**	0.10717	**7.8155**	98.252
MSTRAT	**79**	0.63339	0.10610	7.7361	98.677
PowerCore	80	0.63337	0.30438	7.8010	**100**
Random	**79**	0.61619	0.13179	7.7485	84.202
Raw data	395	0.61614	0.04793	7.7572	**100**

**Table 3:** Core collection results (wheat dataset).

Software	# of samples	MR	Min MR	SH	CV
GenoCore	**65**	**0.61065**	**0.30199**	**11.1482**	**99.018**
Core Hunter	**65**	0.60400	0.25343	11.1257	96.429
MSTRAT	**65**	0.54568	0.21763	10.9978	89.692
Random	**65**	0.54070	0.20502	10.9858	88.153
Raw data	**556**	0.51715	0.10548	10.9053	**100**

**Table 4:** Core collection results and system resources (rice 700K dataset).

Software	Input file size	Used memory	# of samples	CV	Runtime
GenoCore	1.6 Gb	53 Gb	62	99.000	7 h 15 min

If there are only thousands of markers, one rare allele could be located within a block of phenotype-affecting rare alleles. However, high density SNPs and NGS could select multiple alleles as markers within those blocks. Most of previously developed software are focused on one of rare markers because of blindness in those blocks. Our approach assumes that we already know those blocks of rare alleles by high-throughput technologies.

**Fig 5:**
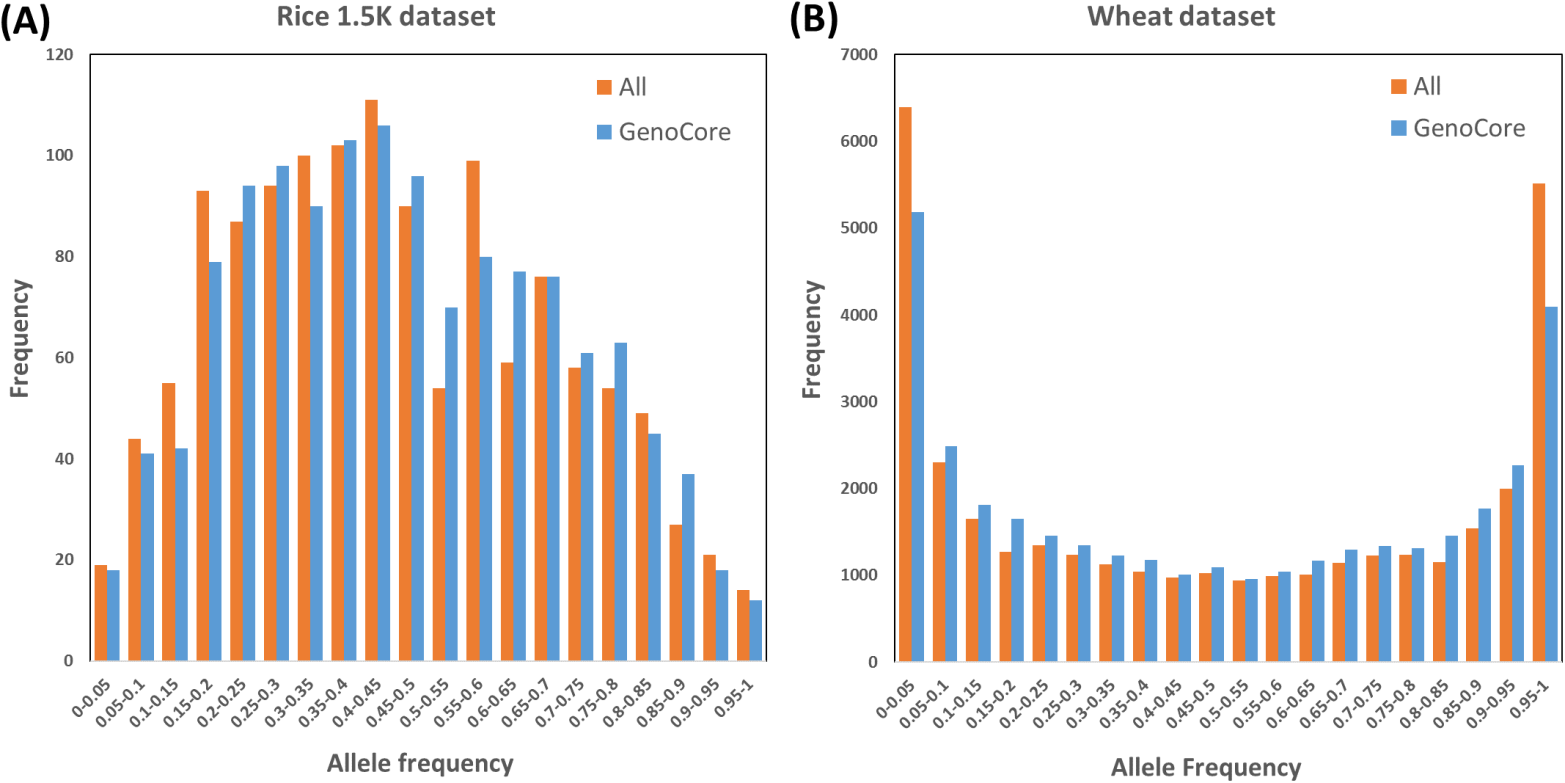
Histogram of allele frequency (rice 1.5k dataset). This is an allele frequency for reference allele of entire and core samples. A and B are the rice 1.5K and wheat dataset, respectively. They have similar distribution.

In case that some samples have many minor allele markers, GenoCore properly selects core collection including those samples. Fig 5 shows that histogram of allele frequency between the entire and core collection in rice 1.5k and wheat dataset (A is the rice 1.5K dataset and B is the wheat dataset). Our method is based on sampling with common alleles, but the result core subset of GenoCore still has a similar distribution for allele frequency of entire samples. Therefore, GenoCore selects core collection controlling the minor allele frequency.

The input file size, computation time, and used memory used for each method are summarized in Tables 5 and 6. The file sizes are different among the methods due to the file formats used to represent multi-alleles. The required memory for each dataset was 4 GB for Core Hunter, and was 80–700 MB for GenoCore, MSTRAT, PowerCore, and random sampling. Although PowerCore used the least amount of memory (80 MB), the runtime was relatively long. By contrast, GenoCore not only used less random access memory but also took less time to execute the procedure.

**Table 5:** System resources (rice 1.5K dataset).

Software	Input file size	Runtime	Used memory
GenoCore	1.1 Mb	<1 min	0.2 Gb
MSTRAT	2.8 Mb	<1 min	0.7 Gb
PowerCore	1.1 Mb	5 min, 40 sec	0.08 Gb
Core Hunter	2.8 Mb	<1min	4.2 Gb

**Table 6:** System resources (wheat dataset).

Software	Input file size	Runtime	Used maximum memory
GenoCore	39 Mb	10 min	1.6 Gb
MSTRAT	130 Mb	10 min	9.7 Gb
Core Hunter	130 Mb	26 min	14 Gb

Core Hunter and MSTRAT conduct random sampling at the first step; therefore, the execution time will be reduced for a smaller dataset, and the results are more consistent with other software. However, these methods have low reproducibility using the default runtime option for large datasets; that is, the selected core samples are quite different for each trial. S1 Fig is a boxplot for the frequency of selected core samples by 100 replicates for each method using the default option in wheat dataset. MSTRAT and Random methods show a low frequency across the selected samples and Core Hunter has a wide spread distribution. The variation in Core Hunter is larger than other methods because it may have stopped the optimization process by the runtime option after random sampling. The higher values the runtime option, the lower the variation. To keep the dispersion close to zero, the runtime option must be more than twice the GenoCore calculation time. However, the frequency of selected core samples by GenoCore is 100 for all replicates. This means that GenoCore provides a consistent core collection for both a low-density SNP chip dataset and a high-density dataset, and even selects the key subset at a faster rate. We could select narrower differences of subsets from those runs if we increase random sampling times in Core Hunter.

## Materials and methods

To develop a new core collection algorithm, we considered two measures that is simple, fast, and consistent to apply to a large dataset. Genotype data is a *m* × *n* matrix that rows are *m* samples, columns are *n* genetic markers, and the type of genotype consists of the four nucleotide bases (A, C, G, and T) or numeric values (representing zygosities for example, 0, 1, 2). Since some samples may have missing genotypes due to experimental errors, these genotypes can be treated as ‘ambiguous’. So, in the first step, candidate samples which have minimum count of missing genotypes are selected and then we calculate the statistical measure of the coverage score for *j*-th sample, denoted by *C*_*j*_. This score means the representativeness of the sample and is defined by

$$C_{j}=\frac{\sum_{i\in N_{j}}f_{ig_{ij}}}{n(N_{j})}$$

where $f_{{ig}_{ij}}$ is the genotype frequency *g*_*ij*_ for the *i*-th marker and *j*-th sample, *N*_*j*_ is the set of nonmissing genotype markers in sample *j*, and *n*(*N*_*j*_) is the number of elements in *N*_*j*_. A sample with high coverage score have more highly genotype frequency markers than other samples. This makes it possible to select samples that have the more common alleles. For this reason, we select the sample having the highest score that makes the pre-defined coverage CV increasing. Because samples with identical coverage scores have the same or similar genotypes, choosing all of these samples reduces the time to total procedure, but it does not help to increase the genetic coverage. Therefore, if more than two samples have the same score, then diversity score is calculated for those samples and we select a sample with the minimum score to select one of those. Nevertheless, it remains samples after above steps, then the core sample is randomly selected. The diversity score means the variability for *j*-th sample, indicates a degree that contains common alleles, and is defined by

$$D_{j}=\frac{\sum_{i\in N_{j}}{\left(f_{ig_{ij}}-C_{j}\right)}^{2}}{n(N_{j})}$$

The coverage and diversity scores measure the extent to which the sample covers the population and the genetic diversity for each sample. For each step, GenoCore selects a sample that is the most representative of the dataset and repeats the process until the coverage reaches 100% (or user can choose the percentage) or the coverage-increasing rate (difference between the coverage of *i*-th step and *(i-1)*-th step) reaches a user-defined threshold (default value is 0.01%). In the case that there are large number of markers or genetically similar samples, the more samples are selected, the lower coverage-increasing rate is.

Choosing a random sampling at the first stage and optimizing the core subset may be a good choice if there are only thousands of marker. However, when the number of markers increase to hundreds of thousands, it takes a lot of time and resources to obtain the core subset after random sampling and the results may vary whenever we calculate. It could give low reproducibility, and not easy to get consistent results from multiple runs. Since we use two simple statistical measures, the coverage and diversity scores, and each iteration reduces the size of data matrix by removing the selected sample and genetically identical samples, our method is capable of fast computation after a lot of iterations. If you have only small number of markers from tandem repeats or restriction fragments, GenoCore may not give better results. However, it will give optimized and consistent subsets for high-density markers.

## Discussion

The key subset from the population is important because this makes experimental cost and time to decrease, but there are a few software for this. In this study, we use an intuitive approach to select a core collection from large, diverse genetic datasets. We define two statistics, the coverage score and diversity score (see Materials and Methods), that are simple and have computational advantage for large datasets. They represent that the average and variance genotype frequency of the sample for all markers. As a result, GenoCore chooses a sample with more common and less variated alleles rather than a sample including a very rare allele when there are millions of markers.

To assess the performance of our method, we use three real datasets (wheat 35k [10], rice 1.5k [6] and rice 700k [9]), three core collection programs (MSTRAT [2], Core Hunter [4], and PowerCore [3], and three measurements. The newly defined coverage score makes the genetic coverage of core subsets to increase faster than other methods (Fig 1) and the diversity score is to correct the bias that can occur in the selection of focusing common alleles. Therefore, the minor allele frequency of our result is similar to that of the entire population (Fig 5). We conduct the principal component analysis to evaluate the position of core collection in the PCA plot of entire samples and confirm that the result of GenoCore is suitably spread (Fig 2). Our method shows a similar or better values for MR, minMR, SH, and CV when compared to the other programs (Tables 2 and 3). Especially, GenoCore has a biggest minMR, this means that the minimum genetic distance between samples in the core collection is the largest. Nevertheless, this does not make the results of GenoCore quite different from those of other methods.

One of the goals in developing new algorithm is to minimize system resource and to be able to calculate for a large dataset, for example, high-throughput array data and whole genome sequencing data. Our method requires less memory and execution time compared to the other core collection software (Tables 5 and 6). Other program cannot be executed for another large dataset, for example, 700k rice SNP chip, 180k soybean SNP chip [8] and whole genome sequencing data (data is not shown). Only GenoCore can be used for large datasets such as high-density SNP arrays and next-generation sequencing, because it is written with R statistical language, which is flexible and has efficient memory. GenoCore can be downloaded from https://github.com/lovemun/Genocore and includes an example.

## Supporting information

S1 Fig Boxplot for reproducibility using 100 replicates. (TIF)
