# Supplementary figures and images for: GenoCore: A simple and fast algorithm for core subset selection from large genotype datasets
Source: PLoS One. 2017 Jul 20;12(7):e0181420. doi: 10.1371/journal.pone.0181420 (PMC5519076; doi:10.1371/journal.pone.0181420)

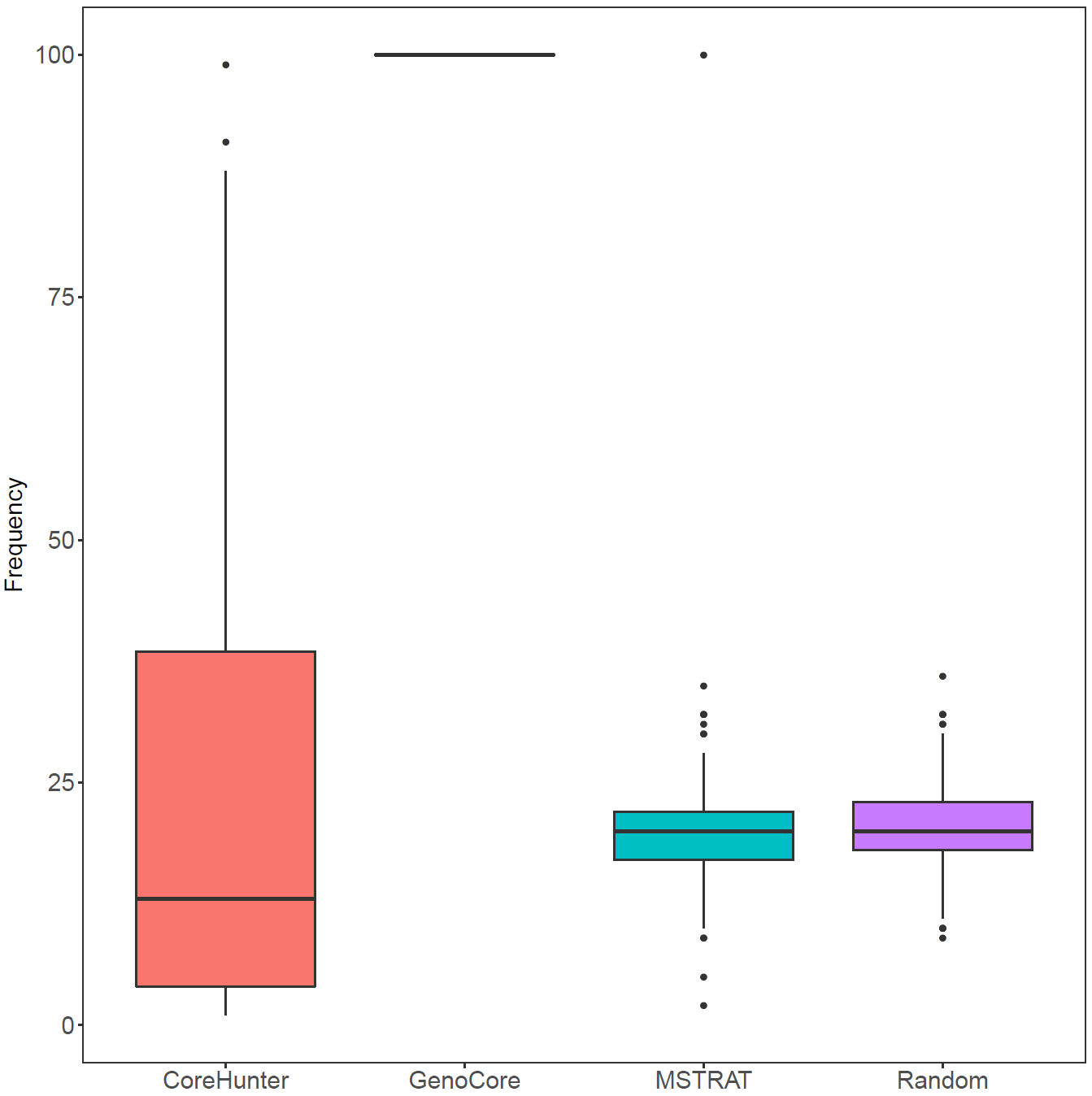

Supplement: S1 Fig — (TIF) [file pone.0181420.s001.tif]
